# Landscape Composition and Management History Affect Alfalfa Weevil but not its Parasitoid

**DOI:** 10.1093/ee/nvac057

**Published:** 2022-08-18

**Authors:** Makenzie E Pellissier, Tatyana A Rand, Melanie A Murphy, Randa Jabbour

**Affiliations:** Department of Plant Sciences, University of Wyoming, Laramie, WY, USA; USDA-ARS, Pest Management Research Unit, Northern Plains Agricultural Research Laboratory, Sidney, MT, USA; Department of Ecosystem Science and Management, University of Wyoming, Laramie, WY, USA; Program in Ecology, University of Wyoming, Laramie, WY, USA; Department of Plant Sciences, University of Wyoming, Laramie, WY, USA; Program in Ecology, University of Wyoming, Laramie, WY, USA

**Keywords:** alfalfa weevil, *Hypera postica*, *Bathyplectes curculionis*, conservation biological control, landscape configuration

## Abstract

It is widely recognized that both local and landscape-scale factors can be important drivers of crop pests, natural enemies, and biocontrol services. However, recent syntheses have found that landscape effects are inconsistent across study systems, highlighting the need for system-specific research to guide management decisions. In particular, studies conducted in perennial crops and that examine landscape configuration, not just composition, are especially lacking. We studied the impact of local and landscape factors on alfalfa weevil *Hypera postica* and its parasitoid *Bathyplectes curculionis*. Although classical biological control efforts have largely suppressed *H. postica* in the eastern United States, it remains problematic in the western United States. We sampled 20 production alfalfa fields in southeastern Wyoming to estimate *H. postica* density, parasitism rates by *B. curculionis*, and vegetation at local scales. We used remotely sensed imagery to characterize both landscape composition and configuration surrounding each sampled field. We used a hypothesis-driven modeling approach to determine which model was most predictive of *H. postica* and parasitism rate by *B. curculionis.* Landscape composition was the best model to predict *H. postica* densities. Host density was the best predictor of parasitism rates by *B. curculionis*. Production fields that had received insecticide applications in the last 5 years had higher weevil densities than fields that had not received insecticide applications. Stand age was not associated with weevil density or parasitism rate. In conclusion, we found local, landscape, and management components to be important in this system.

Ecosystem services like biological pest control vary according to factors across spatial scales (Kremen 2005, Chisholm et al. 2014). Although habitat management of natural and seminatural habitats has long been touted as an effective strategy to support natural enemies (Landis et al. 2000), a recent meta-analysis (Karp et al. 2018) reported the effects of landscape composition on pests and natural enemies are inconsistent and cannot be easily predicted across studies, particularly across studies featuring different crop and landscape features. Natural and seminatural habitats provide enemies with alternative food sources, refuge from disturbance, different microclimates, and overwintering or hibernation sites (Landis et al., 2000). However, cases where more natural habitat in landscapes do not increase natural enemy activity occur (reviewed in Tscharntke et al. 2016), and such habitats potentially provide refuge and resources for pests as well. Rarely do studies examining the impact of landscape extend beyond insect abundance to determine crop damage from pests or crop yield – more relevant metrics for growers (as reviewed by Chaplin-Kramer et al. 2011, Veres et al. 2013, and Karp et al. 2018). In addition, more studies should be cross-scale to examine the relative contributions of local and landscape complexity (Chaplin-Kramer et al. 2019). Conservation of natural enemies may be more attainable in perennial cropping systems, like alfalfa *Medicago sativa* L. (Fabales: Fabaceae), where disturbances are less severe (Landis et al. 2000), however 90% of studies measuring landscape composition effects have been conducted in annual crops (Karp et al. 2018).

In addition to the composition of landcover classes in the surrounding landscape, the configuration of these habitats should be considered to identify conditions necessary to promote biological pest control (Fahrig et al. 2011, Haan et al. 2019). Landscape composition has been the most commonly used metric to describe landscapes by agroecologists (Karp et al. 2018), but configuration has been proposed as a possible explanation for why natural habitat may fail to improve biological control (Tscharntke et al. 2016). Edge density of natural or seminatural habitats near crop fields has been used as a configuration metric in other insect agroecology studies with findings that edge density can be associated with increased insect diversity in crop fields (Holland and Fahrig 2000) and reduced aphid densities (Baillod et al. 2017). Other aspects of configuration that can impact agricultural pests include grain size, shape complexity, and connectivity (as reviewed in Haan et al. 2019).

In our region, alfalfa is a commonly occurring crop and also the site of past classical biological pest control attempts. Alfalfa weevil *Hypera postica* Gyllenhal (Coleoptera: Curculionidae) was first found in the United States in the early 1900’s, and later in the century the United States Department of Agriculture released several parasitoid species to help manage alfalfa weevil (Kingsley et al. 1993). Although some of these parasitoids persist in our region (Brewer et al. 1997, Rand 2013, Al Ayedh et al. 1996), alfalfa weevil continues to be the most problematic pest for farmers (Jabbour and Noy 2017). *H. postica* is a specialist of alfalfa whose densities can vary widely from field to field (Rand 2013). Although no prior alfalfa weevil studies have explored the impact of landscape, we hypothesize that both landscape composition and configuration could be important (Table 1). Given that *H. postica* is a specialist herbivore, we hypothesize its abundance could be associated with the amount of its host plant alfalfa in the surrounding landscape, in line with the resource concentration hypothesis (Root 1973). We also hypothesize that the configuration of alfalfa could predict *H. postica* densities, such that larger patches of alfalfa better support this herbivore. Finally, given documentation of *H. postica* estivating in trees (Manglitz 1958, Prokopy et al. 1967), we hypothesize that density of natural edges, another configuration metric, may be associated with *H. postica* densities.

**Table 1.** Hypotheses for potential predictive factors for herbivore alfalfa weevil and its parasitoid

Hypotheses for alfalfa weevil *Hypera postica*	
Landscape Composition Hypothesis	*H. postica* densities are associated with the proportion of different land covers in the landscape (i.e. lower densities of herbivores in more complex landscape and higher densities of herbivores in landscapes with more host habitat).
Alfalfa Aggregation Hypothesis	*H. postica* densities are positively associated with the aggregation of alfalfa fields in the landscape because dispersal to new fields will be easier.
Natural Edge Estivation Hypothesis	*H. postica* densities are positively associated with the density of natural and seminatural edges in the landscape because these areas are estivation sites.
**Hypotheses for parasitoid *Bathyplectes curculionis***	
Host Density Hypothesis	*B. curculionis*is a specialist parasitoid of alfalfa weevil, therefore parasitism rates will be associated with the population dynamics of its host.
Food Resources Hypothesis	As an adult *B. curculionis* relies on sugary food sources such as aphid honeydew or open blooms. Access to these food sources increase lifespan and fecundity and are therefore positively associated with parasitism rates.
Natural Edge Hypothesis	Parasitism rates are positively associated with the amount of natural edges because they provide more opportunity for parasitoids to enter natural landscapes to access resources.
Natural Edge/Host Density Hypothesis	If *H. postica* are positively associated with natural edges and parasitism rates are negatively associated with host density, then we expect parasitism to be negatively associated with natural edges – because parasitoids are responding to *H. postica* density, and not natural edges themselves.

Similarly, we have a series of hypotheses related to biological control of alfalfa weevil by parasitoid *Bathyplectes curculionis* (Thompson) (Hymenoptera: Ichneumonidae) (Table 2). Aphid density in alfalfa has been positively associated with parasitism rates of alfalfa weevil, likely due to provisioning of parasitoid food resources in the form of honeydew (Jacob and Evans 1998, Rand and Lundgren 2019). Longevity of *Bathyplectes curculionis* was lengthened in the presence of dandelion in laboratory experiments as compared to longevity in the presence of alfalfa (Jacob and Evans 2000). Dandelion was used because it is a common weed in alfalfa, however no published work has examined whether the density of blooming weeds in the field is associated with parasitism of alfalfa weevil. At the local field scale, we hypothesize that parasitism rate may be associated with 1) *H. postica* host density (as in Rand 2013) or 2) food resources such as aphid honeydew and floral nectar. With regards to landscape, given the dispersal capacity of these parasitoids (Evans 2018), we hypothesize that natural edges in particular may be associated with parasitism rate, either given potential for resource provisioning or in interaction with host densities (Table 1).

**Table 2.** Loadings of principle components from principle components analysis at 4 landscape scales

	% Alfalfa	% Other Crop	% Natural/Seminatural
PC1 500 m	0.204	−0.936	0.875
PC2 500 m	−0.959	0.311	0.461
PC1 1,000 m	−0.451	−0.632	0.996
PC2 1,000 m	0.890	−0.773	−0.078
PC1 2,000 m	−0.034	−0.972	0.977
PC2 2,000 m	−0.997	0.234	0.210
PC1 3,000 m	0.099	−0.980	0.975
PC2 3,000 m	−0.990	0.198	0.221

Other in-field characteristics that can influence insect abundance and diversity in alfalfa include weed coverage (Elliott et al. 2002), field age (Fahrig and Jonsen 1998, Zumoffen et al. 2012), and number of physical disturbances within the season (Fahrig and Jonsen 1998). In addition to frequency of physical disturbance and weed management, land managers may influence alfalfa weevil through grazing (Goosey et al. 2004), irrigation (Rand 2013), and raking (Blodgett et al. 2000). Effective insecticide products are labeled for alfalfa weevil control in the United States, but their use varies depending on many factors the producer weighs in making a decision (Jabbour and Noy 2017). We do not know how pesticide application in previous years impacts current insect communities in alfalfa fields.

The objectives of this work were to 1) determine how landscape factors and in-field characteristics affected both abundance of *H. postica* and the activity of its parasitoid *B. curculionis*, 2) determine if pest abundance was associated with alfalfa plant damage, and 3) examine the impact of past pesticide use and alfalfa stand age on *H. postica* density and parasitism rates by *B. curculionis*.

## Materials and Methods

### Field Sites

In 2014, 10 alfalfa production fields were sampled to confirm that parasitoids were still present in our region, given that this was the first survey for weevil parasitoids in Wyoming since 1996 (Brewer et al. 1997). *H. postica* were reared to quantify parasitism using methods described below and parasitism rates of *H. postica* by *B. curculionis* averaged 42.8%, ranging from 13.2 to 74.9%. No other parasitoid species were detected or have been in our other efforts in Wyoming (Rand et al. 2018), thus our work here is focused on *B. curculionis*. In 2015, we sampled 20 alfalfa fields, each separated by at least 4 km from one another to allow for independent analysis of surrounding landscapes. All field sites were located in southeastern Wyoming in Laramie, Platte, and Goshen counties.

Producers were contacted via phone before sampling to determine stand age, variety grown, irrigation type, and general management practices, and to confirm permission to sample their fields. Not all producers were available to answer questions and in some cases producers were unsure and unable to provide information for all questions. Of these, 18 fields were pivot irrigated, and two fields were flood irrigated. Stand age ranged from one to eight years with an average of 3.25 years (*n* = 15, unknown for five fields). Producers were asked if they had applied insecticide to control alfalfa weevil in the last five years. Insecticide had been applied 10 fields sometime in the last five years, five fields had not received insecticide application, and spray history was unknown for five fields. All fields were sampled in June 2015 prior to the first hay cutting or any insecticide application within that season.

Sampling dates were determined based on growing degree days accumulated above 48 degrees Fahrenheit after March 1 to target the third instar larval stage of *H. postica* (Brewer et al. 2008). Fields were sampled after 500 cumulative degree days, and the presence of third instar *H. postica* larvae was confirmed by field visits. Samples were collected from all fields in the narrowest time frame possible (within two weeks) to limit temporal differences in *H. postica* life stages and parasitism rates.

### Insect Sampling

Insects were collected using sweep net sampling (38.1 cm in diameter) on warm, sunny, and low wind days. Six, 50-sweep samples were collected from each field (total of 300 sweeps per field). The first sweep transect began 20 m into the field and was conducted parallel to the field edge. Each subsequent transect began another 10 m into the field (to create a ladder pattern if seen from above). The contents of each 50-sweep sample were transferred into a gallon size bag with a dry paper towel to soak up excess moisture. Gallon bags were transported back to the lab in a cooler for further processing.

To determine parasitism rates of *H. postica* larvae by parasitoids, a subsample of larvae was selected to rear to adulthood. Within twenty-four hours of sampling, one hundred third or fourth instar *H. postica* larvae were randomly removed for rearing. These *H. postica* larvae were divided into two brown paper bags (for a total of fifty larvae in each) with two stems of fresh alfalfa per bag (Al Ayedh et al. 1996). An additional two stems of alfalfa were put in each rearing bag every other day. At the end of 2 wk, the contents of the rearing bags were sorted to determine the number of *H. postica* adults, pupating larvae, and number of parasitoid cocoons (keys provided by W.H. Day USDA-ARS). The remainder of the insects from the sweep sample was frozen until sorted. *H. postica* larvae and adults were counted, as well as aphids (Hemiptera: Aphididae) to include in models to predict parasitism.

### Habitat Characterization

Each field was characterized to account for differences in insect damage, alfalfa height, and floral resources through data collection at the starting location of each of the six sweep transects per field described above. Insect damage to alfalfa was scored by visually inspecting five plants in a 1 m × 1 m quadrat for 10 s and scoring damage on a scale from zero to five, with zero indicating no visible damage and five complete defoliation. Alfalfa height was measured in the middle of each of six the quadrats. Floral resources provided by weeds were quantified along two 50 m transects, each 50 m apart, beginning in-field, and moving towards the field edge. The number of open blooms for each blooming species (excluding alfalfa) was recorded along the transect.

### Landscape Analysis

Landscape composition surrounding field sites was analyzed to determine their relationship to insect abundances and parasitism rates. Latitude and longitude coordinates (decimal degrees, WGS84) were taken with a Global Positioning System (Garmin eTrex 10 Worldwide Handheld GPS Navigator) 20 m into each field site and imported into ArcMap (ESRI, 1992-2014). The area around each set of field coordinates was buffered at four radii: 500 m, 1,000 m, 2,000 m, and 3,000 m. We selected these scales based on the range of scale of response of 3,000 m and smaller for specialist enemies and specialist pests demonstrated in a meta-analysis by Chaplin-Kramer and colleagues (2011). The Cropland data layer from the National Agricultural Statistics Service (2015) was used to determine the proportion of land cover types at each of these diameters for all field sites. We created three land cover categories: natural/seminatural habitat, alfalfa, and other crops (not including alfalfa). Natural/seminatural habitat included undeveloped land cover classes (shrubland, woody and herbaceous wetlands, evergreen forest) and grass/pasture and nonalfalfa hay. Grass/pasture and nonalfalfa hay were included in the seminatural/natural category because, on the spectrum of management and disturbance, these crop types are usually managed minimally without irrigation, chemical inputs, and frequent disturbances. Alfalfa included only the alfalfa cover class. Lastly, other crops included fallow/idle cropland, corn, dry beans, peas, winter wheat, barley, sugarbeet, millet, oats, sunflower, triticale, and sorghum cover classes.

To assess effect of landscape configuration, we used both aggregation index and edge density metrics. Aggregation index (He et al. 2000) is a measure of how clumped the target class is given the frequency of the class. We calculated aggregation index in spatialEco (land.metrics; Evans 2021) for the alfalfa class given *H. postica*’s specialization on alfalfa. Edge density indicates the total length of edge over a given area. We calculated edge density (McGarigal and Marks 1995) of natural and seminatural areas at 500, 1,000, 2,000, and 3,000 m buffers around each point.

Many of the land cover composition proportions were correlated with one another. The proportion of other non-alfalfa crops and natural/seminatural habitat was consistently negatively correlated across scales (500 m: *r* = −0.65, *p* < 0.01; 1,000 m: *r* = −0.57, *p* < 0.01; 2,000 m: *r* = −0.90, *p* < 0.01; 3,000 m scale: *r* = −0.91, *p* < 0.01). Also, at the 500,m scale, the proportion of alfalfa and other crops were negatively correlated (*r* = −0.46, *p* = 0.04) and at the 1,000 m scale, proportion of alfalfa was negatively correlated with natural/seminatural habitat (*r* = −0.52, *p*= 0.02). Due to these strong correlations, we used principal components analysis for these variables to create principal components (PCs) that were independent of one another (Chisholm et al. 2014). The first two PCs of the three created were used as predictors in the multiple regression to represent variation in landscape cover. At the 500 m scale, these two PC’s explained 97.3% (PC1: 63.5%, PC2: 33.8%) of the variation. At the 1,000 m scale, the two PC’s explained 99.7% of the variation (PC1: 59.2%, PC2: 40.5%), 99.9% (PC1: 87.8%, PC2: 12.1%) at the 2,000 m scale, and 99.9% (PC1: 90.1%, PC2: 9.8%) at the 3,000 m scale. In general, PC1 explained variation between %natural/seminatural habitat and %other crops, while PC2 explained variation in %alfalfa. The specific loadings for each scale are presented in Table 2.

### Data Analysis

#### Model Selection to Predict Alfalfa Weevil Density

We used a hypothesis driven modeling approach to identify which factors predict *H. postica* densities. This approach allowed us to compare competing models, both across our hypotheses (Table 1) and across landscape scales. We opted for this approach given that we examined competing hypotheses potentially operating at fine and broader scales. The parameters included in the global model comprised all factors measured in this study including the landscape composition PC1, landscape composition PC2, the aggregation of alfalfa in the landscape (‘alfalfa aggregation index’), the edge density of natural and seminatural areas (‘natural edge density’), in-field floral resources (‘bloom’), and alfalfa height. A subglobal local model and a subglobal landscape model were constructed to compare local (bloom + alfalfa height) and landscape factors (PC1 + PC2 + alfalfa aggregation index + natural edge density), respectively. Then, hypotheses models were constructed using a subset of these parameters (Table 3). The subglobal landscape model and any hypothesis model involving landscape parameters were run for all examined scales (500 m, 1,000 m, 2,000 m, and 3,000 m). All models included alfalfa height as a covariate to account for variation in alfalfa development across fields and the potential impact of canopy height on sweep net sampling (Elliott et al. 2002). Linear models were run using base R (R Core Team 2021). For model selection, best models were determined by lowest AICc value using R package ‘*AICcmodavg’* (Mazerolle 2020). A model with an AICc within 2 of the lowest AICc value was considered comparable. For models containing the landscape factors, the scale with the lowest AICc value was determined to be the most predictive scale.

**Table 3.** Summary of weevil density models and the parameters included in each model

Model	Parameters	Scale	AICc	ΔAICc	Adj *R*^2^
Landscape Composition Hypothesis	PC1 + PC2 + Alfalfa Height	2,000 m	67.36	0	0.57
Landscape Composition Hypothesis	PC1 + PC2 + Alfalfa Height	3,000 m	72.75	5.38	0.44
Null	Intercept	N/A	78.22	10.85	
Natural Edge Estivation Hypothesis	Natural Edge Density + Alfalfa Height	2,000 m	78.32	10.95	0.17
Subglobal: Local	Alfalfa Height + Bloom	N/A	78.69	11.33	0.15
Natural Edge Estivation Hypothesis	Natural Edge Density + Alfalfa Height	1,000 m	78.73	11.37	0.15
Alfalfa Aggregation Hypothesis	Alfalfa Aggregation Index + Alfalfa Height	3,000 m	78.78	11.42	0.15
Natural Edge Estivation Hypothesis	Natural Edge Density + Alfalfa Height	3,000 m	79.52	12.16	0.11
Landscape Composition Hypothesis	PC1 + PC2 + Alfalfa Height	500 m	79.61	12.24	0.21
Natural Edge Estivation Hypothesis	Natural Edge Density + Alfalfa Height	500m	79.75	12.39	0.1
Alfalfa Aggregation Hypothesis	Alfalfa Aggregation Index + Alfalfa Height	2,000 m	79.91	12.54	0.1
Global	PC1 + PC2 + Alfalfa Aggregation Index + Natural Edge Density + Alfalfa Height + Bloom	2,000 m	79.91	12.55	0.53
Alfalfa Aggregation Hypothesis	Alfalfa Aggregation Index + Alfalfa Height	500m	80.44	13.08	0.07
Alfalfa Aggregation Hypothesis	Alfalfa Aggregation Index + Alfalfa Height	1,000 m	80.61	13.25	0.06
Subglobal: Landscape	PC1 + PC2 + Alfalfa Aggregation Index + Natural Edge Density	3,000 m	81.31	13.95	0.26
Subglobal: Landscape	PC1 + PC2 + Alfalfa Aggregation Index + Natural Edge Density	2,000 m	81.4	14.03	0.25
Landscape Composition Hypothesis	PC1 + PC2 + Alfalfa Height	1,000 m	82.43	15.07	0.09
Global	PC1 + PC2 + Alfalfa Aggregation Index + Natural Edge Density + Alfalfa Height + Bloom	3,000 m	83.45	16.09	0.44
Subglobal: Landscape	PC1 + PC2 + Alfalfa Aggregation Index + Natural Edge Density	500m	87.23	19.87	0
Subglobal: Landscape	PC1 + PC2 + Alfalfa Aggregation Index + Natural Edge Density	1,000 m	87.23	19.87	0
Global	PC1 + PC2 + Alfalfa Aggregation Index + Natural Edge Density + Alfalfa Height + Bloom	500m	91.08	23.72	0.18
Global	PC1 + PC2 + Alfalfa Aggregation Index + Natural Edge Density + Alfalfa Height + Bloom	1,000 m	93.59	26.23	0.07

Models with landscape components as parameters include all four landscape scales. The difference in AICc values from the lowest value is indicated by Δ AICc.

#### Model Selection to Predict Weevil Parasitism

A similar approach was used to determine which factors predict parasitism rates of *H. postica*. The parameters included in the global model comprised all factors measured related to parasitism rates including the landscape composition PC1, landscape composition PC2, natural edge density, bloom, alfalfa height, *H. postica* density, and aphid density. As described above, subglobal local, subglobal landscape, and hypotheses models were constructed using a subset of these parameters (Table 4). Generalized linear models with a binomial distribution were used to investigate factors related to parasitism rates using base R (R Core Team 2021). The best model and most predictive scale was chosen using the AICc by the same method described above.

**Table 4.** Summary of models for parasitism rates of *H. postica* and the parameters included in each model

Model	Parameters	Scale	AICc	Δ AICc	Adj *R*^2^
Host Density Hypothesis	Weevil Density	N/A	−11.67	0	0.2
Natural Edge & Host Density Hypothesis	Natural Edge Density + Weevil Density	3,000 m	−10.76	0.92	0.25
Natural Edge & Host Density Hypothesis	Natural Edge Density + Weevil Density	2,000 m	−10.51	1.17	0.24
Natural Edge Hypothesis	Natural Edge Density	2,000 m	−9.99	1.69	0.13
Natural Edge & Host Density Hypothesis	Natural Edge Density + Weevil Density	1,000 m	−9.68	1.99	0.2
Natural Edge Hypothesis	Natural Edge Density	1,000 m	−9.63	2.04	0.11
Natural Edge & Host Density Hypothesis	Natural Edge Density + Weevil Density	500m	−9.44	2.23	0.19
Null	Intercept	N/A	−9.2	2.47	
Natural Edge Hypothesis	Natural Edge Density	3,000 m	−9.2	2.47	0.09
Natural Edge Hypothesis	Natural Edge Density	500 m	−8.95	2.72	0.08
Subglobal: Local	Alfalfa Height + Bloom + Weevil Density + Aphid Density	N/A	−7.93	3.74	0.35
Food Resources Hypothesis	Aphid Density + Bloom	N/A	−5.96	5.72	0.03
Subglobal: Landscape	PC1 + PC2 + Natural Edge Density	1,000 m	−5.58	6.09	0.14
Subglobal: Landscape	PC1 + PC2 + Natural Edge Density	2,000 m	−3.53	8.14	0.04
Subglobal: Landscape	PC1 + PC2 + Natural Edge Density	3,000 m	−2.91	8.77	0
Subglobal: Landscape	PC1 + PC2 + Natural Edge Density	500m	−2.83	8.84	0
Global	PC1 + PC2 + Natural Edge Density + Alfalfa Height + Bloom + Weevil Density + Aphid Density	2,000 m	−1.14	10.54	0.57
Global	PC1 + PC2 + Natural Edge Density + Alfalfa Height + Bloom + Weevil Density + Aphid Density	3,000 m	1.64	13.31	0.5
Global	PC1 + PC2 + Natural Edge Density + Alfalfa Height + Bloom + Weevil Density + Aphid Density	1,000 m	4.84	16.52	0.4
Global	PC1 + PC2 + Natural Edge Density + Alfalfa Height + Bloom + Weevil Density + Aphid Density	500m	7.35	19.03	0.32

Models with landscape components as parameters include all four landscape scales.

#### Damage to Alfalfa Crops

To determine if herbivore densities were associated with alfalfa damage, a linear model was used to test for the effect of *H. postica* larval density and aphid density on alfalfa damage scores.

#### Role of Producer Management

Producers were interviewed to collect data on stand age and past insecticide use (Y/N). These variables were not included in the above modeling approach because these factors were not known for all fields and excluding fields for which these data were unknown would considerably reduce our sample size. We conducted *t*-tests to determine if densities of *H. postica*, aphids, and percent parasitism differed between fields sprayed or unsprayed in the past. Welch’s *t*-test was used due to unequal sample sizes (10 fields sprayed in the past vs 5 fields unsprayed in the past). Linear regression was used to determine if stand age predicted densities of *H. postica*, aphids, or parasitism rates.

## Results

### Model Selection to Predict Alfalfa Weevil Density

**Fig. 1.**
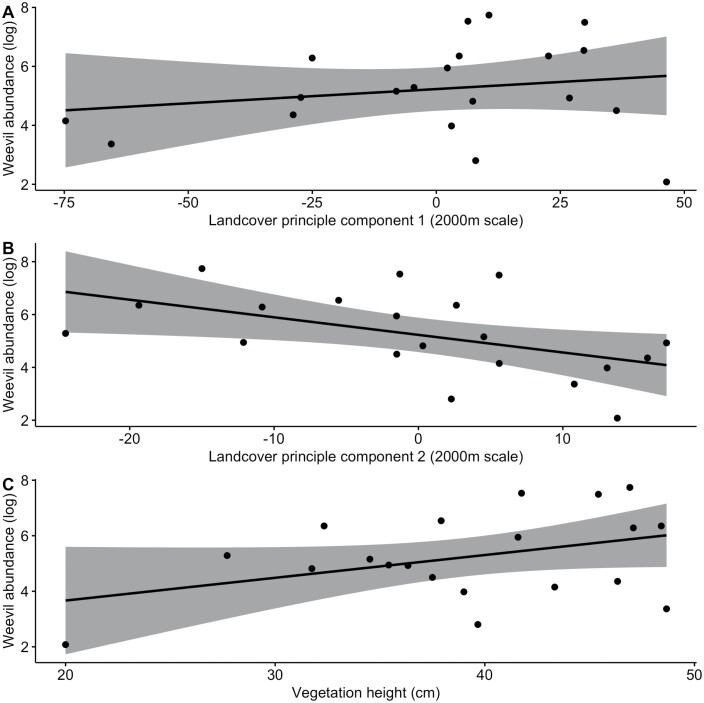
Alfalfa weevil abundance was best predicted by the landscape composition model at the scale of 2,000 m, including the predictors of landcover principal component 1 (A), landcover principal component 2 (B), and the covariate of in-field vegetation height (C). Principal components represented land cover composition categories. PC1 explained variation in % natural/seminatural habitat (positive loading) and % other crops (negative loading). PC2 explained variation in % alfalfa (negative loading). Grey areas represent 95% confidence intervals.

Densities of *H. postica* ranged from 8 to 2,295 larvae per 50 sweeps with a mean (±SE) of 491 larvae (±153) per 50 sweeps. The ‘landscape composition’ model was the best model for predicting *H. postica* densities in alfalfa fields, with the 2,000 m scale the most predictive (Tables 3 and 5). The ‘landscape composition’ model at 3,000 m was the next best model, and the only other one that performed better than the null. The ‘landscape composition’ models included two PCs to describe the landscape and alfalfa height as a covariate (Fig. 1). *H. postica* densities were positively associated with PC1 scores and negatively associated with PC2 scores. Based on the loadings (Table 2), *H. postica* densities increased with natural/seminatural habitat in the landscape and decreased with other crops in the landscape (PC1) and increased with the amount of alfalfa in the landscape (PC2). Lastly, *H. postica* densities were positively associated with alfalfa height. All other models tested for *H. postica* had an AICc that was comparable or greater than the null model (Table 3).

**Table 5.** Summary of results of best fit (lowest AICc) linear models testing various models on *H. postica* density and parasitism rate by *B. curculionis*

Model	Adj *R*^2^	Parameters	Estimate (SE)	*t* value	Pr (>|t|)
*Models to Predict Weevil Density*					
Landscape Composition Hypothesis – 2,000 m scale	0.57	Intercept PC1 PC2 Alfalfa Height	−0.0786 (1.363) 0.0225 (0.0081) −0.0779 (0.0201) 0.1358 (0.0344)	−0.058 2.780 −3.871 3.950	0.955 0.013 0.001 0.001
Landscape Composition Hypothesis – 3,000 m scale	0.44	Intercept PC1 PC2 Alfalfa Height	0.3435 (1.6073) 0.0261 (0.0102) −0.0658 (0.0275) 0.1250 (0.0406)	0.214 2.551 −2.395 3.081	0.833 0.021 0.029 0.007
*Best Model to Predict Parasitism Rate of Alfalfa Weevil*					
Host Density	0.20	Intercept Weevil Density	0.2898 (.0445) −1.23e-04 (5.25e-05)	6.507 −2.344	<0.001 0.032

Models included here were better than the null.

### Model Selection to Predict Parasitism Rates of Alfalfa Weevil

Parasitism rates of *H. postica* ranged from 0 to 56%, with an average parasitism rate of 23% (±4%). The ‘host density’ model had the lowest AICc value, more than 2 AICc lower than the null model (Table 4). Parasitism rates were negatively associated with *H. postica* density (Table 5). The ‘natural edge & host density’ and ‘natural edge’ models at some scales had AICc values within 2 of the ‘host density’ model, but these were also within 2 AICc of the null model (Table 4).

### Damage to Alfalfa Crops

**Fig. 2.**
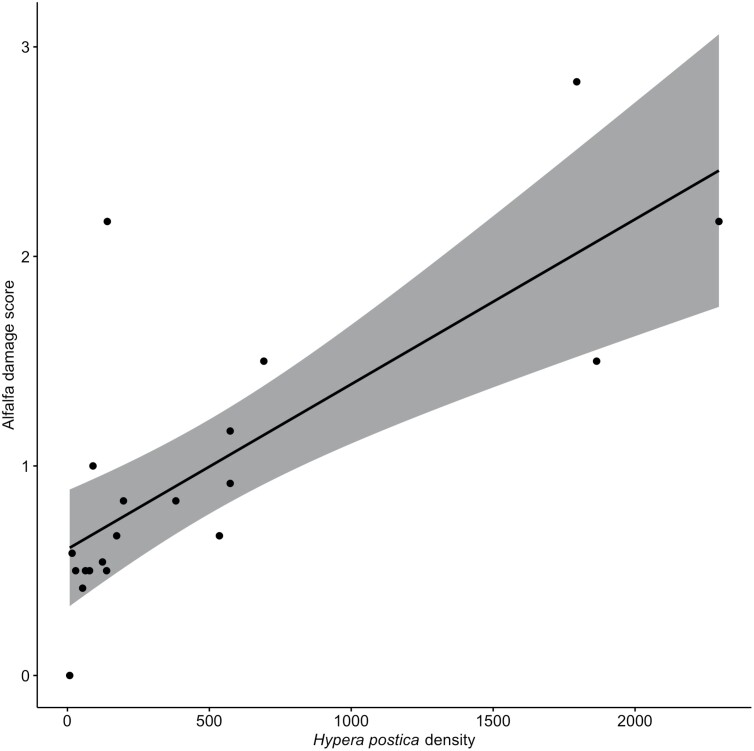
Relationships between alfalfa damage scores and *H. postica* density. Damage was rated at the time of sampling on a scale of zero to five, with zero being no damage and five being complete defoliation. Grey areas represent 95% confidence intervals.

*H. postica* density predicted greater alfalfa damage (Fig. 2, *t* = 4.057, *p* < 0.001). Aphid density was not a significant predictor of alfalfa damage (*t* = −0.37, *p* = 0.72). The overall model had an adjusted *R*^2^ of 0.43.

### Role of Producer Management

Producers reported insecticide use against alfalfa weevil in 10 out of 15 sampled fields in the previous five years, and insecticides were not used in five out of 15 fields. *H. postica* densities were nearly 10 times higher in fields where insecticides had been applied in previous years than those fields that had never been sprayed (753 ± 266 weevils/50 sweeps vs 62 ± 18 weevils/50 sweeps) (*t* = −2.60, *p* = 0.032). Aphid densities (*t* = 1.29, *p* = 0.23) and parasitism rates (*t* = 0.56, *p* = 0.586) did not differ according to past insecticide use. Stand age reported by producers ranged from 1 to 8 years (average of 3.25 years). Stand age was not associated with *H. postica* densities (*R*^2^ = 0.03, *p* = 0.531), aphid densities (*R*^2^ = 0.00, *p* = 0.87), or parasitism rate (*R*^2^ = 0.05, *p* = 0.465).

## Discussion

Our approach helps address identified gaps in conservation biological control research by integrating both local and landscape scales, including landscape composition and configuration (Chaplin-Kramer et al. 2019, Haan et al. 2019). Landscape composition was important in this research, the first time demonstrated for alfalfa weevil. In particular, landscape composition at scales of 2,000 m and larger were associated with alfalfa weevil densities, in line with previously reported predictive scales for specialist herbivores (Chaplin-Kramer et al. 2011). *H. postica* densities were negatively associated with landscape cover of nonalfalfa crops and positively associated with their food crop alfalfa. Specialist herbivores have been shown to be associated with the area of suitable host material in other cropping systems (O’Rourke et al. 2011, Rand et al. 2014), in line with the resource concentration hypothesis that specialist herbivores are more abundant in simple environments with low plant diversity (Root 1973). Given our findings, we suggest land managers consider the strategy of crop diversification across the landscape. O’Rourke and Peterson (2017) highlight mechanisms through which the resource concentration hypothesis could impact pests across landscapes – for instance, dispersal mortality and fitness costs when specialist herbivores must travel long distances to locate their host crop. Many reasons exist to promote crop diversity: provision of floral resources at different time points for both native and honeybees, economic diversification of markets, risk mitigation in the case of extreme weather, and disruption of weed and plant pathogens cycles (Roesch-McNally et al. 2018, Egli et al. 2021). Although alfalfa is a highly marketable crop, growers with major concerns about alfalfa weevil who manage a large area (ideally their surrounding 2 to 3 km), should consider incorporating alternative crops into their management. Although it may be feasible for a single manager of a large operation to make an impact at the landscape-scale required for this pest, promoting cooperation across private landowners to adopt conservation strategies would also be fruitful to explore (e.g., Panchalingam et al. 2019).

In addition to the importance of crops in the landscape, weevil density was positively associated with the proportion of seminatural and natural habitat in the landscape at scales of 2,000 m and 3,000 m. Natural areas may provide estivation sites for alfalfa weevils. In early studies of alfalfa weevil in the eastern United States, weevil adults were recovered from surface litter (under trees, fence rows, and bushes) around the border of alfalfa fields (Manglitz 1958). Weevils estivated in the summer when the weather was hot and dry and returned to alfalfa fields in the fall or in early spring (Prokopy et al. 1967). More estivating weevils were found in borders with straw vs. without straw, demonstrating that residue cover was an important characteristic of estivation sites. In a recent synthesis, exotic pest abundance increased with the proportion of seminatural habitat in the landscape (Tamburini et al. 2020). The authors proposed that benefits of seminatural habitats to exotic pests (i.e., shelter and overwintering sites) may outweigh negative effects that impact native pests – such as more abundant communities of native natural enemies.

In our work, no model predicted parasitism rate by *B. curculionis* better than host density. Similarly, Rand (2013) found that host density was a significant driver of parasitism rates of *H. postica*, although the relationship differed over the two years of her study. Parasitism rates may be lower in fields with higher *H. postica* densities if *B. curculionis* simply cannot keep up with heavy *H. postica* infestations (Al Ayedh et al., 1996), and these relationships may change between years depending on the population dynamics. At higher host densities, parasitism can be constrained by egg-load and handling time of hosts, resulting in parasitoids not being able to fully take advantage of all available hosts (Hassell and Waage 1984). Although we found no association between aphid density and weevil parasitism rate, findings from both Montana and Utah demonstrate the importance of aphid honeydew as a resource for *B. curculionis* (Evans and England 1996, Rand and Lundgren 2019). Rand and Lundgren (2019) found a positive association between aphid density and parasitism in 2 out of 5 years of their study. Our findings may be limited by our research occurring only in a single field season, particularly given the potential for host-parasitoid population cycles operating over multiple years. No landscape composition effects on parasitism rate were documented in our study, possibly in part due to abundant in-field resources provided to parasitoids through both weevil hosts and aphid honeydew (Tscharntke et al. 2016). In addition, Evans (2018) demonstrated that parasitism rates of alfalfa weevil did not change with distance into a newly planted alfalfa field, suggesting rapid dispersal of these parasitoids into new habitats. Nevertheless, several models including natural edge density had similar AICc values as our best model to predict parasitism (Table 4), suggesting the potential for impacts on weevil-parasitoid interactions.

Our work supports the assertion from others (Chaplin-Kramer et al. 2019, Paredes et al. 2021) that variables relevant to management must be more thoroughly incorporated into agroecology pest management research. We found that management history – specifically, whether insecticide was applied in the previous 5 years – was tied to weevil densities, with higher densities in fields with history of application. Perhaps these fields have consistently high weevil pressure so are more likely to be sprayed, or it could signal possible development of insecticide resistance, documented in neighboring state Montana (Rodbell and Wanner 2021). Given the importance of landscape composition to predict weevil densities, we examined the landscapes surrounding these fields with history of insecticide application. These fields were located in landscapes with more alfalfa (21.1% landcover) in contrast to fields with no application in recent years (6.4% alfalfa landcover) at the scale of 2,000 m. We are limited in our ability to interpret the role of past pesticide use given our lack of detail about pesticide applications and spray history being confounded with landscape in this data set. Future research could tease apart these factors by sampling from paired fields within the same landscapes, with or without past pesticide use, along a gradient of % alfalfa landcover. In addition, following fields for multiple years after a spray event, could provide additional perspective on how a legacy of insecticide use impacts insect communities in the future.

In conclusion, our snapshot into alfalfa weevil-parasitoid interactions revealed that alfalfa weevil densities were best predicted by landscape composition at the 2,000 m scale and parasitism rates were best predicted by host density. Landscape configuration did not play a major role in predicting weevil density or parasitism rate. Future work that explores how weevil estivation relates to natural habitats, pairs fields with and without insecticide application history across landscape gradients, and studies the same fields over multiple years would be valuable next steps to understanding how to limit damage from this important pest.
